# Progress in Surgery

**Published:** 1894-02-24

**Authors:** 


### PROGRESS IN SURGERY.

### SKIN GRAFTING.

Thiersch's skin grafting is now so universally employed by practitioners as well as surgeons, with a host of skilled assistants, that any modification of this method is of interest. The following technique has been found by Dr. C. L. Gibson[1] to be very satisfactory. The ulcer and the part to yield the graft are rendered absolutely aseptic for a week before operation. The skin is shaved, washed thoroughly, and macerated for twelve hours with a layer of green soap spread on muslin and covered with oiled paper, then scrubbed well with a brush, washed with equal parts of alcohol and sulphuric ether, and finally with corrosive sublimate (1 in 1,000). The wet sublimate dressing is renewed daily. The whole process is repeated two days before operation, and the part irrigated with normal saline solution. All granulations are scraped away from the ulcer, the edges pared, bleeding completely stopped, and sterilised compresses applied wet with the saline solution. The patient being placed under ether, the grafts are cut with a hollow-ground razor from the extensor surface of the thigh; they are cut from above downwards in strips two inches wide and five or six inches long. The razor should not penetrate the subcutaneous tissue, and the motion should be even and rapid. The grafts are floated, raw surface uppermost, in warm saline solution on to strips of protective tissues, their frayed edges trimmed off, and then applied to the ulcer, the strips being very close to each other. The grafts are then covered with strips of protective tissue one and a half inch wide, overlapping each other. Sterilised gauze dressing kept wet by saline solution through a number of drainage tubes between its layers affords the necessary moisture. The dressings are removed at the end of a week, and reapplied after gentle irrigation of the grafts. For large chronic ulcers of the leg, and in plastic operations on the face, especially after extensive operations for lupus or epithelioma, transplantation of large skin flaps without a pedicle has recently been introduced. The method is employed for cases in which Thiersch's grafting is inapplicable. In Krause's experience[2] of twenty-one cases only four suffered complete necrosis of the flap. He takes only the cutis and cuticle without the subcutaneous connective tissue, and the size seems to have no importance provided the operation is done aseptically. The flap should be folded wound surface against wound surface to avoid unnecessary manipulation. It is never necessary to suture it to the surrounding tissue. In a fresh wound or after scraping or excision of an ulcer the whole field is washed with sublimate solution, then with salt solution, and afterwards thoroughly dried with sterilised gauze. Ligatures should never be used to check hæmorrhage as they act as foreign bodies. The skin to be utilised is carefully prepared in the same way, but strong scrubbing with brushes should be avoided.

As a dressing Krause uses sterilised iodofor gauze firmly bandaged over the wound with fixation of the limb. The hands of the operator and instruments should be thoroughly dry. The dressing should be changed on the third or fourth day, the whole limb being soaked in boracic solution for an hour or more. Four cases of transplantation of skin flaps without a pedicle, but containing the subcutaneous fatty tissue, are reported by Hirschberg[3]. The operations were undertaken for defects after the extirpation of tumours. In three cases the bleeding was controlled by compression, but in one, two fine catgut ligatures had to be applied. Both the wet and dry bandages after transplantation frequently result in failure of firm adhesion or tearing away or loosening of some pieces which subsequently necrose. Mayer[4] bridges over the wound and surrounding surface with a thin piece of wood or firm pasteboard, or Gooch's splinting resting on pillows of wool or cotton placed one above and the other below the surface operated on. A starch bandage is applied externally. At the elbow or dorsum of foot strips of plaster of Paris modelled appropriately may be used, and likewise resting on the pillows. Large skin grafts after excision of lupus have recently been used by Watson Cheyne[5] with success in the case of a lad, the whole cheek being thus renovated.

### HERNIA.*

In certain severe and obstinate cases of hernia all methods of radical cure are open to failure. Rushton Parker[6] in the discussion at the August meeting of the British Medical Association dealt with the means employed by himself. His herniotomies up to date numbered 291 in nineteen years, and out of 250 operations for radical cure 61 were strangulated and 190 unstrangulated; in the latter there were eight deaths, or 4·2 per cent., attributable to the operation. Ligature of the sac high up, whether inguinal or femoral, yielded gratifying results; the sac was stripped from the spermatic vessels and duct, but in scrotal cases only enough was stripped up to get hold of and enable its offset from the peritoneal pouch to be reached—the part below was left to take care of itself. Among causes of failure may be noted flabbiness or distension of abdomen and insufficient enforcement of the recumbent posture. Parker keeps his patients lying six weeks at least after herniotomy. Improved antiseptic practice and the use of silk ligatures are important factors. From his experience Parker concludes that the radical cure of inguinal hernia in childhood and infancy can be attained with ease and almost certainty. The cure of inguinal and femoral hernia in men and women is attainable in the large majority, the exceptions being sometimes large, sometimes small herniæ. He believes that Macewen's operation is the best for inguinal and femoral hernia at all periods of life. Ward Cousens[7] believes that the sac cannot always be excised in congenital cases—80 per cent. of his cases (120) varying from one to twelve years were successful by one operation. The sac was never opened if empty; it was drawn down after being deeply separated, and secured by two ligatures half an inch apart. Finally the plug formed by the ligatures was fixed in the canal by another ligature passed through the tendinous structures above the ring.

The very decided distrust of attempts at reduction by taxis in strangulated hernia and the preference for immediate operation is the subject of an article in Mr. Hutchinson's archives[8]. He traces the changes which have taken place in opinion, the results within his own experience, and the employment of Petit's operation. As regards his own personal experience of force in applying taxis he says, "I can only say that I used all that my hands possessed, and that I often wished that they were stronger and did not tire so soon." Although the soft parts were often ecchymosed as a result of bruising, yet he never saw anything in the gut itself attributable to the manipulation, and had not known the occurrence of rupture of the intestine. He believes that private cases are less hopeful than those seen in hospital.

For the avoidance of suppuration in radical cure, the most minute care is necessary on the part of the surgeon and of all who have to do with the operation. Heat and chemicals are relied upon for disinfection and asepsis. C. B. Lockwood[9] uses carbolic acid (1 in 40) for the instruments and biniodide or perchloride of mercury for the rest. After the patient has had a hot bath, the part is carefully shaved and washed with soft soap and water, and its sebaceous matter removed by ether. Perchloride or biniodide of mercury in alcohol (1 in 1,000 for the former and 1 in 2,000 for the latter) is next applied, and then carbolic gauze dressing—the layers nearest the skin being soaked in glycerine of perchloride (1 in 2,000) or biniodide of mercury and glycerine (1 in 4,000), or in one part of carbolic to 40 parts of glycerine. A very large area of the abdomen is treated in this way. Biniodide of mercury is used for the sponges, and a solution of perchloride of mercury in spirit (1 in 500) for the hands of the operator and assistants after they have been thoroughly cleansed. The wound is disinfected, closed, dusted with iodoform, and covered with carbolic gauze soaked in carbolic or other weak disinfectant. A strap and buckle round the thigh and another round the pelvis with a spica bandage over all keeps the dressing from being displaced. Eighty-five per cent. of Lockwood's non-strangulated cases healed by primary union.

### IN FEMORAL HERNIA.

Lockwood transfixes the neck of the sac after separation and secures it with a stout silk ligature tied in a Staffordshire knot. The ends of the ligature are passed in turn upon a Macewen's needle up the femoral canal along the sub-peritoneal tissue and through the abdominal wall about the middle of Hesselbach's triangle, just above the inguinial canal. The skin and subcutaneous fat being drawn aside, the silk is tied in a firm knot upon the surface of the aponeurosis of the external oblique. The common femoral vein is protected with the finger during the passage of the needle up the canal. The neck of the sac should now be within the abdomen, and the femoral canal quite empty; this is now closed by fastening together Hey's and Cooper's ligaments by means of a strong silk ligature passed with a Macewen's needle. The contents are treated in the usual way. Lockwood has never seen suppuration around the omental pedicle such as Lucas-Championnière describes in some of his cases[10]. Fatal peritoneal hæmorrhage has been caused by tearing some of the omental vessels near the stomach whilst pulling the omentum down. In twelve non-strangulated cases (two males and the rest females) ten healed by first intention, in one only death occurred on the sixth day from bronchitis after ether. One dressing was enough for each. Lockwood obtained a proper radical cure five times after kelotomy for strangulated femoral hernia, four healing by first intention under a single dressing. Bell[11] operated upon a right femoral hernia which contained a strangulated vermiform appendix and a mass of omentum. In the centre of the latter there was a cavity resembling the interior of the large intestine. The appendix and omentum were removed and the cæcum returned, the patient making a good recovery.

### INGUINAL HERNIA.

The operation for radical cure of inguinal hernia is confined by Lockwood to the congenital herniæ of young adults, the age averaging 18 years eight months. In congenital hernia there is no morbid defect of the peritoneum and mesentery, but only a local developmental defect of the abdominal wall, capable of being completely remedied by operation. Children are not very suitable for operation, but it should be undertaken if the rupture is small or a truss cannot be worn. Inguinal herniæ in little girls give excellent results. He divides the operations into two classes—(1) those in which the inguinal canal was not opened; (2) those in which the canal was freely opened by division of the aponeurosis of the external oblique. In his procedures he avails himself of the operations of Mitchell Banks, Macewen, Ball, Barker, Kocher, Bassini, and Halstead. The scrotum should be avoided, but this is not possible in large strangulated herniæ. The "ordinary" inguinal operation as described by Lockwood was performed 17 times in males; 14 of these healed by first intention, one had very slight superficial suppuration, and in two suppuration led to the extrusion of some of the deep sutures. The inguinal canal was laid open, and Bassini's operation performed in nine male cases; two of these had sub-acute suppuration, one slight stitch-hole suppuration, and the remaining six healed by first intention under a single dressing. Of seven female cases operated on for radical cure of congenital inguinal hernia, six healed by first intention (one with two dressings), and one suppurated on the sixth day, due to the presence of staphylococcus aureus in the skin. Of 44 recoveries after radical cure of non-strangulated hernia, 36 healed directly, of these 30 required a single dressing, five required two, and one, a troublesome schoolboy, required several. Of the remaining eight, five had suppuration with extrusion of deep sutures, and three had very slight suppuration. Although there is the greatest difficulty in tracing cases, yet the ultimate results of many of Lockwood's operations, both inguinal and femoral, show that the radical cure is as succesful as any operation in surgery.

An oblique inguinal hernia in a man aged 32, operated on by Bell by Macewen's method, is probably unique. It was omental and congenital, and the unusual feature which explained the impossibility of retaining it by a truss proved to be a hydatidiform cyst (cyst of Morgagni) growing from the omentum and adherent to the bottom of the sac of the tunica vaginalis. Another congenital hernia in a boy aged three contained cæcum and ilium adherent to the cord. In both recovery was uninterrupted after operation with no return of the hernia. Suppurative inflammation of the sac of an inguinal hernia in a youth aged seventeen, which had been produced by prolonged and ineffectual manipulations, simulated strangulation in another of Bell's cases.

### Hernia of Ovary.

Two cases of inguinal hernia of the ovary, one of which was situated in the labium majus, were operated upon by C. B. Lockwood[12]. In both muscular tissue was found; in the first case at the back of the sac, and in the second connecting the end of the sac with the labium. A hernia of the ovary and tube in an active condition of tubercular disease was successfully operated upon by Bell[13]. It had come through the inguinal canal of an infant aged twelve months.

### Umbilical Hernia.

The umbilical hernia of adults is certainly capable of radical cure. For the radical cureof large umbilical herniæ, Gersuny[14] brings the exposed margins of the recti muscles into immediate apposition by sutures. After the omentum and sac have been removed in the usual manner, and the peritoneal cavity closed, the sheaths of the recti are exposed at their inner margins by being cut away. The muscle is then separated from its sheath by the knife on each side, in the space between the two nearest lineæ transversæ. The external wound being very extensive, primary union is not expected; the superficial sutures are left until some days later. Two cases of successful radical cure of congenital umbilical hernia on the second and fourth days respectively after birth, are described by Berger[15]. Both were instances of hernia into the cord, the structures of which formed the covering, the contents consisting of the cæcum, appendix, and colon were irreducible, and adherent to the wall limiting the hernial cavity. Laparotomy was performed, adhesions carefully divided, intestine reduced, and the abdominal wall closed by three rows of sutures. Berger thinks that no operation should be undertaken when the arrest of development of the abdominal wall is so great as to render complete closure impossible, or in puny children or those before full term. Guéniot, in the discussion on one of Berger's cases, referred to Lindfor's monograph "On Umbilical Hernia," which appeared last January in Volkmann's Vorträge. Three cases of ventral hernia, treated by radical operation, are recorded by M'Ardle[16]. After operation, want of union of the mid-stratum, made up of the elastic fascia of Scarpa, and the aponeurosis formed by the fused abdominal tendons—the only pathological condition present—was caused by (1) failure to engage the different layers of this stratum sufficiently in the sutures; (2) interposition of contused peritoneum; (3) hæmatoma not becoming soundly organised; (4) suppuration from inherent or extrinsic causes.

[1] New York Med. Journal, Aug. 5, 1893; and Sheffield Med. Journal, Oct., 1893. [2] Verhand. der Deutsch. Gesellschaft für Chirurg. xxii, Kongress, 1893; Annals of Surgery, Oct., 1893. [3] Verhand. der Deutsch. Gesellschaft für Chirurg. xxii. Kongress, 1893; Annals of Surgery, Oct. 1893, p. 458. [4] N.Y. Med. J., Nov. 11, 1893, p. 569. [5] Brit. Medical Journal, Dec. 9, 1893, p. 1274. [6] Brit. Med. Journal, Nov. 11, 1893. p. 1037. [7] Brit. Med. Journal, Nov. 11, 1893, p. 1039. [8] Archives, of Surgery, Oct., 1893. [9] Lancet, Nov. 25, 1893, p. 1297. [10] Cure Radicale des Hernies, Paris, 1892. p. 448. [11] Montreal Medical Journal, Nov., 1893, p. 348. [12] Brit. Med. Journal, Dec. 9, 1893, p. 1272. [13] Montreal Med. Journal, Nov., 1893. [14] Centralbl. f. Chir., No. 43. 1893; Brit. Med, Journal, Nov. 18, 1893. [15] Revue de Chir., Oct., 1893. [16] Brit. Med. Journal, Dec. 16, 1893, p. 1328.

